# Ultra Deep Sequencing of Circulating Cell-Free DNA as a Potential Tool for Hepatocellular Carcinoma Management

**DOI:** 10.3390/cancers14163875

**Published:** 2022-08-11

**Authors:** Mónica Higuera, Elena Vargas-Accarino, María Torrens, Josep Gregori, María Teresa Salcedo, Joan Martínez-Campreciós, Gloria Torres, María Bermúdez-Ramos, Itxarone Bilbao, Mercedes Guerrero-Murillo, Xavier Serres-Créixams, Xavier Merino, Francisco Rodríguez-Frías, Josep Quer, Beatriz Mínguez

**Affiliations:** 1 Liver Cancer Research Group, Liver Diseases, Vall d’Hebron Institut de Recerca (VHIR), Vall d’Hebron Barcelona Hospital Campus, 08035 Barcelona, Spain; 2 Department of Medicine, Campus de la UAB, Universitat Autònoma de Barcelona (UAB), Bellaterra, 08193 Cerdanyola del Vallès, Spain; 3 Viral Hepatitis Research Group, Liver Diseases, Vall d’Hebron Institut de Recerca (VHIR), Vall d’Hebron Barcelona Hospital Campus, 08035 Barcelona, Spain; 4 Centro de Investigación Biomédica en Red de Enfermedades Hepáticas y Digestivas (CIBERehd), Instituto de Salud Carlos III, 28029 Madrid, Spain; 5 Pathology Department, Hospital Universitario Vall d’Hebron, Vall d’Hebron Barcelona Hospital Campus, 08035 Barcelona, Spain; 6 Spanish Biomedical Research Network Centre in Oncology (CIBERONC), Instituto de Salud Carlos III, 28029 Madrid, Spain; 7 Hepatobiliary Surgery and Transplant Department, Hospital Universitario Vall d’Hebron, Vall d’Hebron Barcelona Hospital Campus, 08035 Barcelona, Spain; 8 Radiology Department, Hospital Universitario Vall d’Hebron, Vall d’Hebron Barcelona Hospital Campus, 08035 Barcelona, Spain; 9 Biochemistry and Microbiology Department, Hospital Universitario Vall d’Hebron, Vall d’Hebron Barcelona Hospital Campus, 08035 Barcelona, Spain; 10 Biochemistry and Molecular Biology Department, Campus de la UAB, Universitat Autònoma de Barcelona (UAB), 08193 Bellaterra, Spain; 11 Liver Unit, Hospital Universitario Vall d’Hebron, Vall d’Hebron Barcelona Hospital Campus, 08035 Barcelona, Spain

**Keywords:** liquid biopsy, cfDNA, HCC, biomarkers

## Abstract

**Simple Summary:**

In this unicentric prospective study, we analyzed the most prevalent mutations in HCC (TERT promoter, TP53, CTNNB1, AXIN1 and ARID1A) in plasma cfDNA by next-generation sequencing, aiming to elucidate their value as prognostic noninvasive biomarkers. Total cfDNA (cut-off value 2 ng/µL), number of mutated genes and number of detectable mutations on cfDNA were significantly related to mortality. Number of mutated genes and number of detected mutations in cfDNA and the ratio between number of mutations and total amount of cfDNA were also significantly associated with recurrence. Detection of more than four mutations in cfDNA correlated with a higher risk of death. Dynamic changes in cfDNA mutations were detected prior to radiological detection of HCC recurrence. We believe that these results support the proof of principle and launching of validation studies to confirm that total cfDNA and detection of prevalent HCC mutations could have prognostic implications in early-stage HCC patients.

**Abstract:**

Background: Cell-free DNA (cfDNA) concentrations have been described to be inversely correlated with prognosis in cancer. Mutations in HCC-associated driver genes in cfDNA have been reported, but their relation with patient’s outcome has not been described. Our aim was to elucidate whether mutations found in cfDNA could be representative from those present in HCC tissue, providing the rationale to use the cfDNA to monitor HCC. Methods: Tumoral tissue, paired nontumor adjacent tissue and blood samples were collected from 30 HCC patients undergoing curative therapies. Deep sequencing targeting HCC driver genes was performed. Results: Patients with more than 2 ng/µL of cfDNA at diagnosis had higher mortality (mean OS 24.6 vs. 31.87 months, *p* = 0.01) (AUC = 0.782). Subjects who died during follow-up, had a significantly higher number of mutated genes (*p* = 0.015) and number of mutations (*p* = 0.015) on cfDNA. Number of mutated genes (*p* = 0.001), detected mutations (*p* = 0.001) in cfDNA and ratio (number of mutations/cfDNA) (*p* = 0.003) were significantly associated with recurrence. However, patients with a ratio (number of mutations/cfDNA) above 6 (long-rank *p* = 0.0003) presented a higher risk of recurrence than those with a ratio under 6. Detection of more than four mutations in cfDNA correlated with higher risk of death (long-rank *p* = 0.042). Conclusions: In summary, cfDNA and detection of prevalent HCC mutations could have prognostic implications in early-stage HCC patients

## 1. Introduction

Hepatocellular carcinoma (HCC) is ranked as the seventh most common neoplasm and the fourth leading cause of cancer death worldwide. HCC is the most frequent primary liver cancer and is the leading cause of death among patients with cirrhosis [1].

Tumor biopsy remains the standard diagnostic procedure for cancer diagnosis. However, in HCC, biopsy has a limited role in clinical practice because, in cirrhotic patients, noninvasive radiological criteria are well established and validated, leaving biopsy as an indication for patients without cirrhosis or for those patients with cirrhosis with tumors not showing the specific radiological features [2].

Most of the reported knowledge regarding molecular features, molecular classification and potential biomarkers of HCC has been developed using surgical samples from patients at initial stages of the disease. However, tumorigenesis is a dynamic process and new molecular features different from the ones present at initial-stage disease progress over time. Tumor resistance to therapy could occur during treatment, possibly due to newly acquired molecular features that were not present in the initial biopsy sample [3]. Nevertheless, in the era of molecular oncology, where many treatments for different neoplasms are guided by molecular alterations, in clinical practice, the most used tools to classify patients, allocate optimal treatments and estimate prognosis of HCC patients are exclusively clinical algorithms [4].

In this context, despite tumor biopsies still representing the standard procedure for diagnosis and molecular testing to guide precision therapies, liquid biopsy and, in particular, cell-free DNA (cfDNA) from plasma is rapidly emerging as an important and minimally invasive alternative to standard tumor biopsies [5]. Liquid biopsy is a valuable tool for cancer detection and monitoring during and after treatment, and it has been demonstrated to be useful for molecular characterization [6]. cfDNA could also be beneficial in overcoming the problem with tumor heterogeneity, usually under-represented in small or partial tumor samples [7]. Liquid biopsy has shown advantages over conventional tissue biopsy due to its minimally invasive nature, which reduces the risk of complications for patients [8]. Moreover, it is also easily repeatable during patient follow-up [9], allowing monitoring of potential changes at the molecular level. These factors underline the importance of developing liquid biopsy techniques for HCC, considering cancerous cells from this tumor adapt to pharmacological pressure and acquire new molecular alterations that may have been missed or not detected at initial diagnosis [10].

cfDNA concentrations in plasma of HCC patients are up to 20 times higher than in healthy people [11]. Furthermore, cfDNA concentration has been found to be associated with tumor size, portal vein invasion and inversely correlated with prognosis and shorter overall survival [11,12,13]. The potential applications of cfDNA analysis and its mutations also include the detection of potential molecular targets for acquired resistance, uncover potential targets not detected in small biopsy samples or the detection of minimal residual disease after surgical resection [14,15].

There are limited reports on cfDNA in HCC, but recent publications described the detection of HCC-associated driver genes, such as TP53, CTNNB1 and TERT in cfDNA [16,17,18,19,20]. Several of these studies have investigated a small number of patients, reporting only the mutation landscape at the moment of resection with no derived information about the relationship between mutations found and risk of recurrence and patient outcomes.

The aims of this study were to (1) elucidate whether mutations found in plasma cfDNA could be confidently detected using next-generation sequencing, evaluating if they were representative of the most commonly described driver mutations present in HCC tissue, and (2) provide the proof of principle to further develop this technology to monitor the evolution of those mutations in HCC patients during their follow-up. To address these questions, we prospectively recruited 30 HCC patients at early stages undergoing curative therapies and synchronously collected tumor, surrounding liver tissue and whole blood samples, in which deep sequencing targeting HCC driver genes and mutation hotspots was performed.

## 2. Materials and Methods

### 2.1. Study Design and Participants

All patients were recruited at the Liver Unit, Hospital Universitari Vall d’Hebron and prospectively enrolled in the study. Samples from 30 patients with confirmed HCC, either with noninvasive radiological criteria or histological confirmation, were prospectively collected. Matched blood and tissue (HCC and surrounding nontumoral liver) samples were collected simultaneously from these 30 patients receiving curative treatments with surgical resection (*n* = 27) or local ablation (*n* = 3). Historical samples from 2 of these patients, obtained prior HCC diagnosis, were retrieved for this study. Serial blood samples were subsequently collected at multiple follow-up time points. Ten samples from healthy adults were obtained from blood donors from the Blood and Tissue Bank (Banc de sang I teixits, BST, Barcelona, Spain). Additionally, 21 blood samples from HCC patients at intermediate and advanced stages of the disease (BCLC B, *n* = 7; BCLC C, *n* = 8; and BCLC D, *n* = 6) were also prospectively collected.

**Figure 1:**
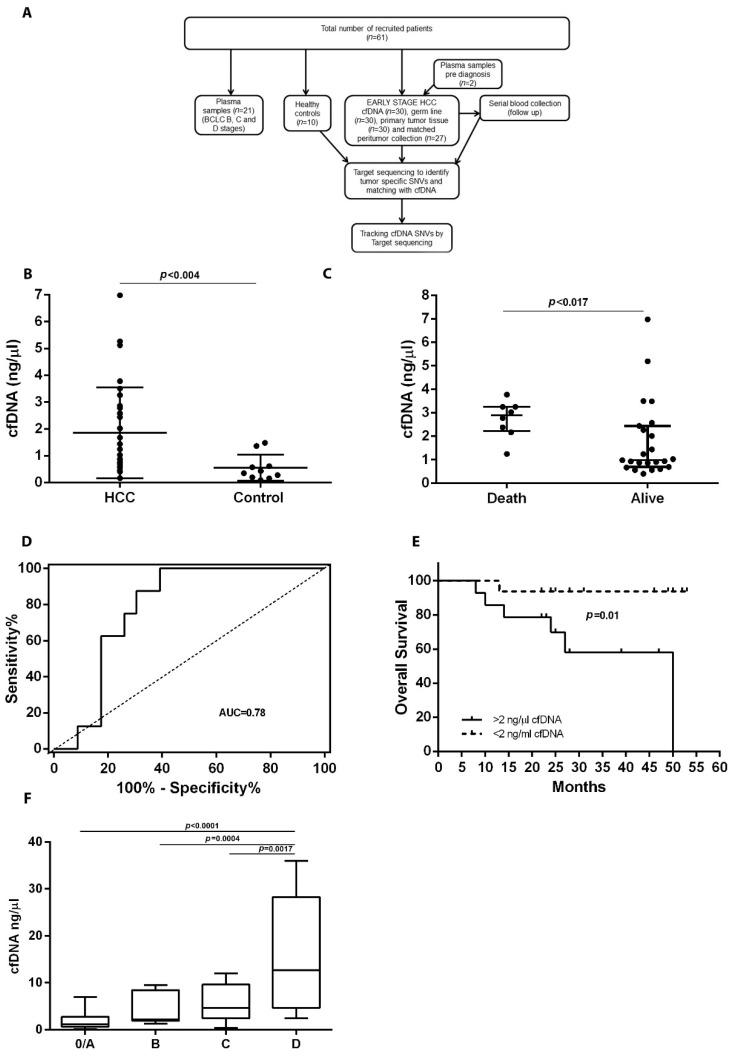
(**A**) Flow diagram of the study design. (**B**) cfDNA concentrations in patients with early-stage HCC and healthy controls. (**C**) cfDNA concentrations in patients with early-stage HCC who were dead or alive at the end of follow-up. (**D**) ROC curve distinguishing patients with more than 2 mutations. (**E**) Kaplan–Meier curve of overall survival for HCC patients stratified by baseline cfDNA level; *p*-value from the log-rank test. (**F**) cfDNA levels stratified by BCLC stage (0: Very early, A: Early, B: Intermediate, C: Advanced and D: End-stage), cfDNA: cell-free DNA.

The detailed study design is shown in Figure 1A. In total, 57 tissue (30 HCC tissues and 27 matched surrounding liver tissues) and 113 blood samples (83 plasma samples to extract cfDNA and 30 samples of peripheral blood mononuclear cells (PBMC) to extract germ line) were collected. The study was conducted in accordance with the Declaration of Helsinki. The institutional ethical review board approved the protocol (PR(AG)194/2015), and all patients gave written informed consent before inclusion.

### 2.2. Sample Collection

Peripheral venous blood was collected at least within the 24–48 h prior to surgery in a lithium heparin tube (BD Biosciences, Franklin Lakes, NJ, USA) and processed within 4 h of collection. Plasma was collected after a first centrifugation at 1600× *g* for 15 min at 4 °C, and then was further centrifuged at 16,000× *g* for 10 min at 4 °C and was immediately stored at −80 °C. Liver specimens were collected ad hoc for this study at the operation room and brought to the Pathology Department to be processed with the help of an expert pathologist for specific tissue sampling and immediately stored at −80 °C.

### 2.3. DNA Extraction and Quantification

Circulating DNA was isolated from 1 mL of plasma using the MagMAX™ Cell-Free DNA Isolation Kit (Thermo Fisher, Waltham, MA, USA). Blood and tissue DNA was isolated using the QIAamp DNA Mini Kit (Qiagen, Hilden, Germany) according to the manufacturer recommendations. Purified DNA concentration was measured by fluorometric quantitation using Qubit (Thermo Fisher, Waltham, MA, USA).

### 2.4. Primer Design and PCR

Primers were designed to amplify different regions enriched with hotspot containing frequent mutations in TERT promoter, TP53, CTNNB1, AXIN1 and ARID1A (Supplementary Materials Table S1). PCR reactions and conditions were performed with the Start High Fidelity PCR system dNTPack (Roche Applied Science, Penzberg, Germany) following manufacturer recommendations, adding 5 μL of template DNA (Supplementary Materials Table S1). PCR products were then subjected to 15 cycles of a universal MID PCR using FastStart Taq DNA polymerase (Roche Applied Science, Penzberg, Germany) as previously published [21]. The final MID amplification yielded from 185 to 216 bp fragments (Supplementary Materials Table S1). The PCR products were analyzed by 2% agarose gel electrophoresis, purified with DNA clean-up (NZY tech, Lisbon, Portugal) and quantified by fluorometric quantitation using Qubit (Thermo Fisher, Waltham, MA, USA). Amplicon quality was analyzed using a BioAnalyzer DNA 1000 LabChip (Agilent, Santa Clara, CA, USA) prior to sequencing using Illumina MiSeq platform (Illumina, San Diego, CA, USA).

### 2.5. Library Preparation and Next-Generation Sequencing

For the sequencing using the MiSeq platform (Illumina, San Diego, CA, USA), amplification products were pooled and purified using Kapa pure beads (Roche Applied Science, Penzberg, Germany). Pools were quantified by Qubit (ThermoFisher, Waltham, MA, USA). The genomic libraries were processed following the manufacturer instructions for DNA library preparation kit Kapa Hyper Prep kit (Roche, Applied Science, Penzberg, Germany) and indexed using SeqCap Adapter Kit A/B (Roche, Applied Science, Penzberg, Germany). The final library was quantified by LightCycler 480 (Roche, Applied Science, Penzberg, Germany) and sequenced using MiSeq sequencing platform with MiSeq Reagent kit v2 (2 × 150 bp mode with the 300 cycle kit) (Illumina, San Diego, CA, USA).

### 2.6. Data Analysis

The raw fastq files acquired from MiSeq were first submitted to FLASH [22] to overlap the paired-end reads and reconstruct full amplicons. Sequencing data analysis was conducted as previously published [21]. An overlapping of paired ends (2 × 300) with a minimum of 20 overlapping bases, and a maximum of 10% differences were established. Full reads carrying 5% or more bases below a Q30 Phred score were discarded. The third step was a demultiplexing by specific amplicon primers with a maximum of three differences. Reads were then collapsed into haplotypes with corresponding frequencies. All haplotypes with abundances below 0.1%, and not common to both strands were discarded and, finally, we filtered all variants below an abundance of 1% [21,23,24].

Raw sequencing data from samples included in this article will be openly available upon publication via Sequence Read Archive of the NCBI (accession number PRJNA791805).

### 2.7. Statistical Analysis

Quantitative clinical variables were described using mean ± standard deviation or median (interquartile range (IQR)) as appropriate to distribution. For qualitative variables, frequency and percentage were calculated. Boxplots by main outcomes have been constructed. Associations between cfDNA levels and clinical outcomes were assessed by nonparametric Mann–Whitney U-test. An ROC analysis was performed to identify cfDNA concentration capacity to discriminate patients with more than two mutations. Kaplan–Meier survival curves were estimated for survival and recurrence by cfDNA level. Hazard ratios and their 95% confidence intervals were calculated from univariate Cox regression for clinical and mutation-related variables. Data analysis was performed in R version 4.1.0 and Stata 15.1. Statistical analysis has been carried out by Statistics and Bioinformatics Unit (UEB), Vall d’Hebron Research Institute (VHIR).

## 3. Results

### 3.1. Study Design and Patient Characteristics

We prospectively profiled blood and tissue samples from 30 patients with early HCC receiving curative therapy and 10 healthy controls. Matched blood and fresh frozen tissue samples were available for the 30 HCC patients. The median follow-up was 22.5 (1–50) months. At least one follow-up sample was analyzed for every patient (Figure 1A). The clinical and demographic parameters of these patients are summarized in Supplementary Materials Table S2. The median age of HCC patients was 61.5 years, 76.6% were male, and liver cirrhosis was present in 26.6% of patients; viral etiology was observed in 43.2% and 16.6% presented metabolic-associated fatty liver disease (MAFLD). The median diameter of the largest tumor was 3.75cm (range, 1–12 cm), while microvascular invasion (mVI) was present in 20% of the tissue samples. During study follow-up, tumor recurrence was observed in 12 patients (40%) and 7 patients died (23.3%).

### 3.2. Quantification of cfDNA in HCC Patients

Median level of plasma cfDNA was significantly higher in HCC patients compared to control subjects (1.73 ng/µL, IQR 0.87–3.08 and 0.38 ng/µL, IQR 0.18–0.79; *p* = 0.004) (Figure 1B). Median cfDNA levels at diagnosis were significantly higher in patients who died during follow-up (*n* = 7) than in patients remaining alive by the end of follow-up (2.90 ng/µL, IQR 2.23–3.26 and 0.98 ng/µL, IQR 0.70–2.44; *p* = 0.0174) (Figure 1C). To investigate the correlation between the concentration of preoperative cfDNA and survival, a cut-off value of 2 ng/µL of cfDNA was established by an ROC curve analysis with AUC = 0.782 (Figure 1D). Patients presenting with greater than 2 ng/µL of cfDNA had a higher mortality compared to patients with less than 2 ng/µL (mean survival time 24.6 months vs. 31.87 months, *p* = 0.01) based on Kaplan–Meier’s survival analysis (Figure 1E). No correlation was observed between cfDNA concentration and tumor size, AFP levels or presence of vascular invasion.

Additionally, cfDNA was quantified in patients with intermediate and advanced HCC (*n* = 7 BCLC B, *n* = 8 BCLC C and *n* = 6 BCLC D) to compare cfDNA levels along BCLC stages. The clinical and demographic parameters of these patients are summarized in Supplementary Materials Table S3. As expected, those patients at more advanced stages (BCLC C and D) had a higher median cfDNA level (5.58 ng/µL, IQR 2.42–9.63 and 15.80 ng/µL, IQR 4.65–28.25, respectively) compared to patients at earlier stages (BCLC 0/A) (1.85 ng/µL, IQR 0.65–2) (Figure 1F).

### 3.3. Mutations Identified in Plasma cfDNA and Matched HCC Tissue DNA

After observing detectable plasma levels of cfDNA in patients with HCC, NGS analysis was initially performed in early HCC tumor samples, matched surrounding tissue, cfDNA and PBMCs (as a germ line) from the 30 HCC patients who underwent curative treatments (resection *n* = 27 or local ablation *n* = 3); samples were analyzed by NGS with a median read depth of 46,281×. In total, 202 nonsynonymous somatic single-nucleotide variants (SNVs) with at least 1% of frequency were identified in the four types of samples analyzed from each HCC patient (*n* = 30). Among all identified SNVs, 87% of them (174/202) were reported in COSMIC database [25], meaning that their occurrence had been previously observed, and 13% (28/223) were novel mutations (Supplementary Materials Table S4).

**Figure 2:**
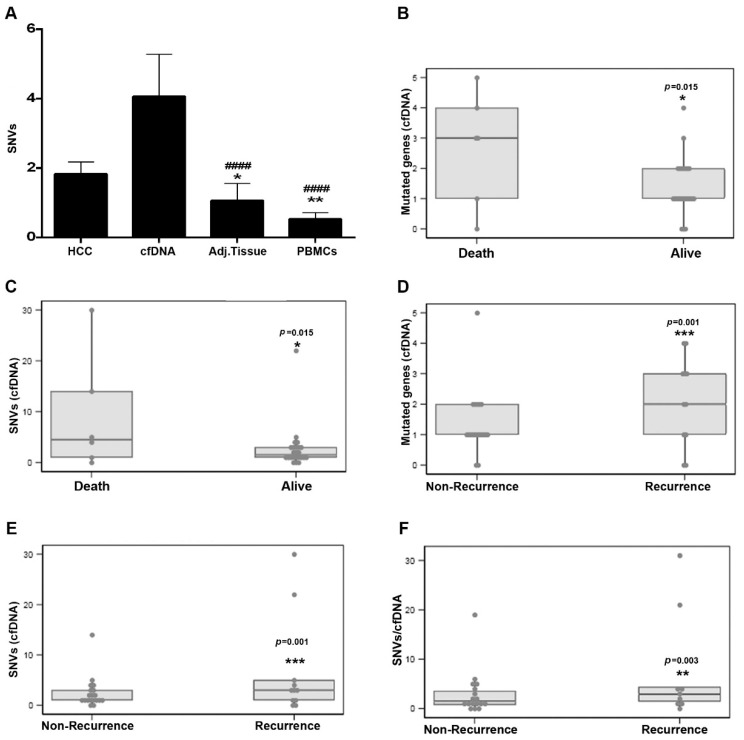
(**A**) Number of somatic mutations detected in HCC tissue, cfDNA, surrounding liver tissue and PBMCs of early-stage HCC patients, * *p* < 0.05, ** *p* < 0.01, *** *p*<0.001 vs. HCC tissue. #### *p* < 0.0001 vs. cfDNA. (**B**) Number of mutated genes detected in the cfDNA and (**C**) number of SNVs detected in the cfDNA of patients with early-stage HCC according to survival status (i.e., death vs. alive). Number of mutated genes (**D**), SNVs detected in the cfDNA (**E**) and ratio SNVs/cfDNA. (**F**) in early-stage HCC patients according to presence/absence of recurrence. cfDNA: cell-free DNA, SNV: single-nucleotide variant, PBMCs: peripheral blood mononuclear cell.

The majority of patients had at least one mutation in HCC tissue DNA and cfDNA (70.6%—24/30; 86.7%—26/30, respectively). An average of 1.9 (range 0–9) mutations per patient were detected in HCC tissue, 4 (0–30) in cfDNA, 1.1 (0–10) in paired-adjacent tissue and 0.4 (0–3) in PBMCs (Figure 2A).

Concordance between genetic variants, both in HCC tissue DNA and cfDNA samples, was analyzed. A total of 55 mutations were found in HCC tissue DNA. Among them, 29 (52.7%) also had evidence of identical carcinogenic mutations in matched cfDNA. Importantly, additional mutations not present in HCC tissue DNA were found in cfDNA. From a total of 104 mutations detected in cfDNA, 75 were additional mutations over those described in HCC tissue DNA. Therefore, mutations identified in cfDNA could provide additional molecular information about the tumor.

We further evaluated whether the number of mutated genes or the number of mutations in cfDNA of patients with early HCC could be useful in predicting prognosis. Subjects who died during follow-up had a significantly higher median number of mutated genes on their cfDNA at baseline than those subjects who remained alive by the end of follow-up (3, 1–4 vs. 1, 1–2; *p* = 0.015) (Figure 2B and Table 1). Patients who died were also more likely to have detectable mutations in their cfDNA (4.5, 1–14 vs. 1.5, 1–3; *p* = 0.015) (Figure 2C and Table 1). The number of mutated genes (Figure 2D) (*p* = 0.001) and detected mutations (Figure 2E) (*p* = 0.001) in cfDNA were also significantly associated with recurrence, as well as the ratio between number of mutations and total amount of cfDNA (number mutations/cfDNA), which was also significantly associated with recurrence of patients with early HCC (1.5, 0.8–3.6 vs. 2.9, 1.4–4.4; *p* = 0.003) (Figure 2F and Table 1).

**Figure 3:**
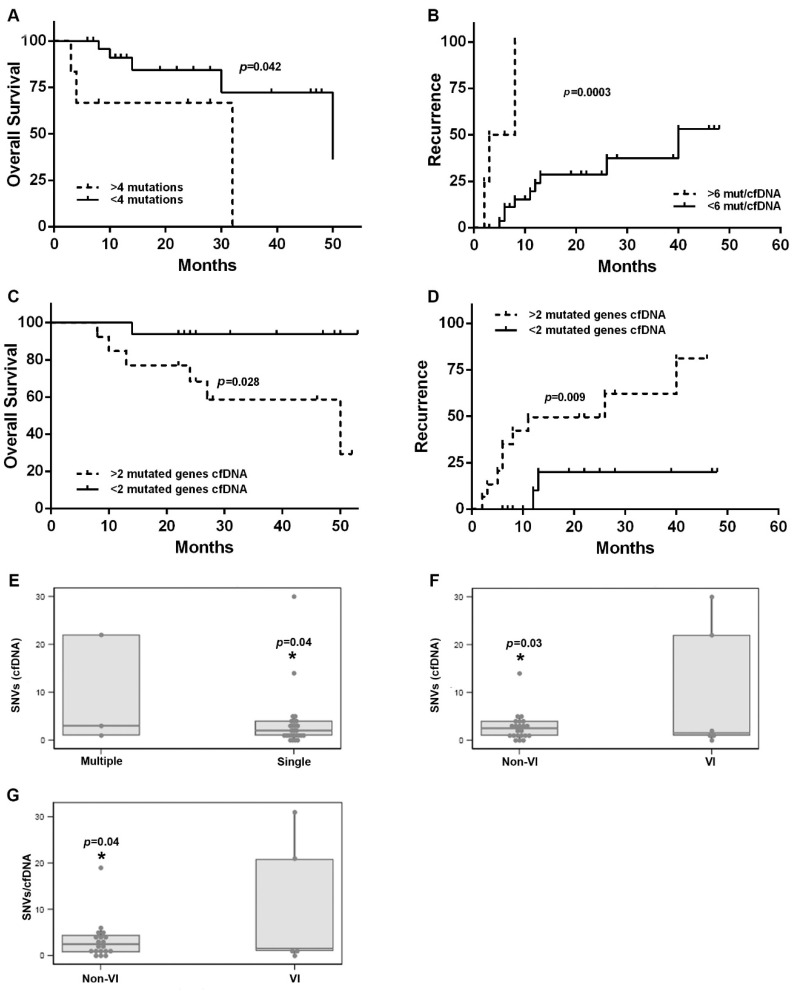
(**A**) Kaplan–Meier curve of overall survival for HCC patients stratified by number of mutations in the cfDNA. *p*-value from the log-rank test. (**B**) Kaplan–Meier curve of recurrence for HCC patients stratified by the ratio of number of mutations/cfDNA. *p*-value from the log-rank test. (**C**) Kaplan–Meier curve of overall survival for HCC patients stratified by number of mutated genes in cfDNA. *p*-value from the log-rank test. (**D**) Kaplan–Meier curve of recurrence for HCC patients stratified by number of mutated genes in cfDNA. *p*-value from the log-rank test. (**E**–**G**) Correlation between SNVs with poor prognosis status: number of SNVs detected in the cfDNA of early HCC patients with single or multiple foci of HCC (**E**), number of SNVs detected in the cfDNA of HCC patients with or without vascular invasion (**F**) and ratio of SNVs/cfDNA (**G**) detected in early HCC patients with or without vascular invasion. SNV: single-nucleotide variant, cfDNA: cell-free DNA, VI: vascular invasion. * *p* < 0.05.

The multivariate analysis showed that detection of more than four mutations in cfDNA correlated with a higher risk of death (long-rank *p* = 0.042) (Figure 3A and Table 2). Those patients with a ratio (number of mutations/cfDNA) higher than six presented a higher risk of recurrence than those with a ratio under six mutations/cfDNA (long-rank *p* = 0.0003) (Figure 3B and Table 2). Patients who presented mutations in more than two genes showed a higher risk of death and recurrence (long-rank *p* = 0.028 and *p* = 0.009, respectively) (Figure 3C,D and Table 2).

Next, we sought to assess the relation between mutational status of cfDNA with well-known poor prognosis factors in clinical practice, observing that those patients with more than one HCC nodule had a higher ratio (number of mutations/cfDNA) (6, 1–31.3 vs. 1.7, 0.8–4.0; *p* = 0.04) (Figure 3E). Moreover, both the number of mutations detected in the cfDNA and the ratio (number of mutations/cfDNA) were observed to be significantly associated with the presence of microvascular invasion (*p* = 0.03 and *p* = 0.04, respectively) (Figure 3F,G). Among clinical and analytical parameters included in Supplementary Materials Table S5, only size of the main nodule was found to be an independent risk factor of tumor recurrence.

### 3.4. Variant Characteristics in Plasma and HCC Tissue

**Figure 4:**
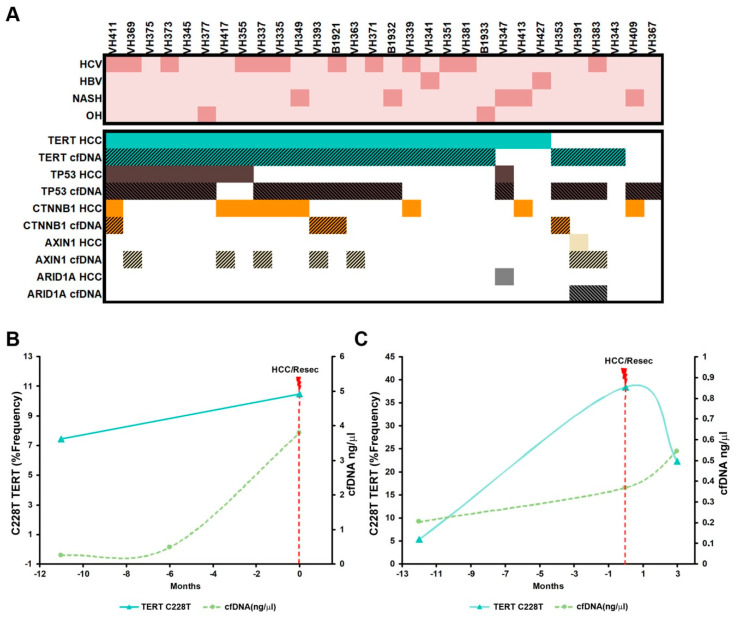
(**A**) Mutational landscape of patients with early-stage HCC using mutations found in cfDNA and HCC tissue in 30 patients. The heatmap illustrates the nonsynonymous mutations detected in plasma cfDNA and HCC tissue and the etiology information of the 30 HCC patients at the time of the curative intervention. (**B**,**C**) Early detection of C228T TERT mutation (−124) before HCC diagnosis in two patients. The activating TERT promoter mutation C228T was detected in the cfDNA 11 and 12 months before diagnosis in VH341 and VH381 patients, respectively. HCV: hepatitis C virus, HCB: hepatitis B virus, NASH: nonalcoholic steatohepatitis OH: alcohol, cfDNA: cell-free DNA.

Further analysis of mutations showed that the most commonly mutated gene in the total HCC patient cohort was TERT. At least one mutation of TERT promoter was found in the 76.7% (23/30 patients) in both cfDNA and HCC tissue. The most common mutation detected in TERT promoter (C228T) was also validated by ddPCR, obtaining a detectable and similar mutation rate in all samples where C228T mutation was detected by sequencing (Supplementary Materials Table S6). At least one mutation in TP53 gene was detected in 50% (15/30) of cfDNA samples versus 33.3% (10/30) of HCC tissue, and CTNNB1 was mutated in 10% of cfDNA (3/30) and 33.3% (10/30) of HCC tissue of patients. Finally, 16.7% (5/30) and 10% (3/10) of patients presented mutations in AXIN1 on their cfDNA or HCC tissue, respectively, and mutations in ARID1A were found in 10% (3/30) and 6.7% (2/30) of patients in cfDNA and HCC tissue (Figure 4A).

### 3.5. Early Detection of Mutations in Driver Genes Prior to HCC Diagnosis

Very few studies have focused on the evaluation of cfDNA detection in early-stage cancers (BCLC 0/A) with even less data available on the detection of ctDNA in pre-HCC-diagnosis stored blood samples from HCC patients. We had the opportunity to analyze cfDNA from previously stored samples from two HCC patients. Samples were collected months before radiological diagnosis of HCC. Samples were analyzed in order to identify potential driver mutations detectable before the radiological diagnosis of HCC. One TERT mutation was found in the cfDNA of patients VH341 (HBV-related HCC) and VH381 (HCV-related HCC) 11 and 12 months before HCC diagnosis, respectively (Figure 4B,C).

Two mutations were detected in the cfDNA from plasma obtained 11 months before the radiological diagnosis of HCC in patient VH341. The activating TERT promoter mutation C228T was detected at a frequency of 8% 12 months before diagnosis and 10% at the time of diagnosis (Figure 4B). Tumor tissue frequency of C228T mutation was 1.95%. R249S mutation in TP53 was also detected at a frequency of 1.65% and 1.4% in cfDNA 11 months before diagnosis and at the time of diagnosis, respectively. The R249S variant was detected at a frequency of 0.4% in tumor tissue.

The activating TERT promoter mutation C228T was also detected 12 months before diagnosis in patient VH381 at a frequency of 5.34%, being 38.4% by the time of HCC diagnosis. Tumor tissue frequency of C228T mutation was 37.72% (Figure 4C).

### 3.6. Dynamics of cfDNA and Mutations during HCC Progression

Next, to further explore whether cfDNA and SNVs dynamically change along with clinical evolution of patients, we analyzed sequential plasma samples collected from our cohort during their clinical course.

**Figure 5:**
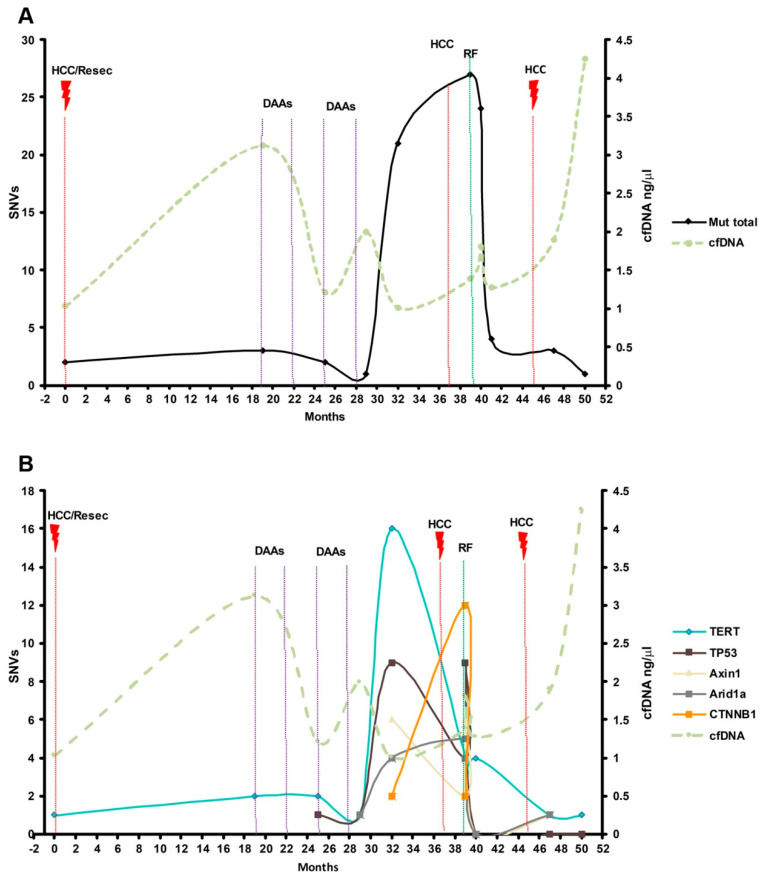
(**A**) Dynamical changes in cfDNA levels and absolute mutational load correlating with HCC progression (Patient VH335). (**B**) Dynamical fluctuations in cfDNA levels and number of mutations in the five driver genes tested along the 52 months of follow-up. HCC/Resec: detection and surgical resection of HCC, DAAs: HCV treatment with direct-acting antivirals, RF: radiofrequency ablation, cfDNA: cell-free DNA, SNVs: single-nucleotide variant.

As shown in Figure 5A, as an example, patient VH335 showed dynamical changes in SNV number and cfDNA levels correlating with HCC progression. Before receiving surgical treatment, low levels of cfDNA (1.03 ng/µL) were quantified and only the C228T in TERT promoter was detected in both cfDNA and HCC tissue (Figure 5B). After 31 months of follow-up with no visible tumor lesions by MRI, cfDNA levels increased to 1.99 ng/µL, and a total of 37 mutations were detected in the cfDNA distributed along the five genes tested: TERT (16), TP53 (9), AXIN1 (6), ARID1A (4) and CTNNB1 (2) (Figure 5B). Radiological progression was diagnosed 37 months after diagnosis and the patient was then treated with radiofrequency ablation. Both cfDNA levels (from 1.81 to 1.27 ng/µL) and observed tumor SNVs decreased after that therapeutic intervention, when four SNVs were detected in TERT promoter. Finally, after 45 months of follow-up, the patient progressed to an advanced HCC, increasing cfDNA levels to 4.25 ng/µL (Figure 5).

Dynamic changes in cfDNA and SNV frequency of another three HCC patients have been included as supplementary data (Figures S1 and S2). Patient VH343 presented fluctuation in cfDNA levels and TERT mutation C228T (−124) correlating with HCC progression and tyrosine kinase treatment (Figure S1a). Moreover, patient VH369 showed dynamical changes in cfDNA levels, which were increased before second HCC relapse (16 months) (Figure S1b). Patient VH371 presented an increase in cfDNA levels and in the number and frequency of mutations months before radiological detection of relapse, 11 months after surgery (Figure S2).

## 4. Discussion

Real-time monitoring of cfDNA levels and mutational burden for patients with HCC has been proposed as a potential tool to improve early diagnosis of HCC and early detection of recurrence after treatment. Recently, the use of cfDNA levels and its molecular analysis has been reported to provide useful information about tumor burden and prognosis by genetic and epigenetic analysis [16,18,26,27].

In this exploratory and prospective study, we investigated the usefulness of cfDNA collected at the time of diagnosis and before curative intervention (resection or local ablation) for quantification and molecular profiling in early-stage HCC patients. Patients at early stages, mostly candidates to surgical resection were selected, aiming first to evaluate the concordance between cfDNA and tumor tissue mutations and, second, to assess dynamic changes in cfDNA after potentially curative treatment to determine its potential value as a biomarker. The selection of patients at early stages defines a very homogeneous population to study cfDNA value, but, at the same time, it is less likely to capture events such as HCC recurrence or death during follow-up to estimate the prognostic value of cfDNA in a short unicentric cohort. We considered this design optimal to explore potential biomarkers to be thereafter validated in a larger cohort. Even with this small sample size, the data are very promising.

Early-stage HCC levels of cfDNA were significantly higher than cfDNA levels in healthy controls, and, as described in prior reports, the highest cfDNA levels were detected in patients with more advanced disease, as a likely consequence of greater tumor cell burden and cfDNA release [19,28]. More interestingly, we found a baseline cut-off value of 2 ng/µL (AUC = 0.782) able to discriminate patients with high and low mortality during follow-up, suggesting that just the quantitative amount of detectable cfDNA could have a prognostic value.

We specifically targeted the most significantly mutated genes and regions in HCC (TERT promoter, TP53, CTNNB1, AXIN1 and ARID1A), evidencing that high-depth sequencing analysis of plasma-derived cfDNA could be used to detect tumor-related gene mutations in plasma cfDNA. Analyzing paired samples of plasma cfDNA and HCC tissue DNA in this cohort, a consistency of types of genes mutated detected in both types of samples of 52.7% was demonstrated, similar to the concordance reported in other studies [7,17]. Our results suggest that most prevalent mutations in HCC identified in the cfDNA are representative of those present in the HCC tumor tissue.

We observed that a high number of variants in plasma could not be confirmed in tumor samples (104 mutations found in cfDNA vs. 55 mutations found in HCC tissues), suggesting that cfDNA could be more informative at a molecular level than small biopsy/surgical under-representative samples. As previously reported, RNAseq studies from different distant regions within the same tumor have evidenced differences in transcription factor signaling [3]. This could explain the fact that finding more mutations in cfDNA than in tumor tissue samples defines the complex molecular heterogeneity in HCC [29]. Subclones could be localized in a different topographic location in the primary tissue [30,31,32], and coexistence of different subclones with distinctive mutational profiles and different spatial location could have considerable practical implications when extracting molecular information from biopsies or partial surgical samples.

The most frequently mutated gene in plasma was TERT promoter, with a frequency of 76.7% (23/30 patients), followed by TP53 mutated in 50% (15/30) of cfDNA samples, consistent with it being described in the previous literature about the mutational profile of early-stage HCC [7,33,34].

Despite the small number of patients included in our study, we have found that the mutational load, defined as the total number of variants detected in cfDNA and the number of mutated genes were associated with overall survival and recurrence in a univariate analysis. In our cohort, interestingly, patients presenting four or more mutations in cfDNA at baseline had shorter survival. Accordingly, a higher number of mutations detected in cfDNA were also associated with well-described poor prognostic factors in the HCC setting, such as the presence of multiple foci of HCC or the presence of microvascular invasion at pathology exam. Interestingly, while those variables, cfDNA number of mutations and genes mutated, showed association with patient outcomes, prognostic clinic-pathological parameters commonly used in clinical practice, such as number of nodules, presence of microvascular invasion or alpha-fetoprotein levels, showed no association with recurrence or overall survival in our cohort. This is probably a consequence of the small number of patients evaluated, all of them at early stages of the disease, being less likely to develop events. These findings provide the proof of principle to test this approach in a larger multicentric cohort of plasma samples of patients at early stages to validate the potential value of our findings.

cfDNA alterations evidenced from our early HCC samples could also be tested in at-risk patients with liver cirrhosis under ultrasound screening programs, given the easy access to blood samples, without needing invasive procedures. In this study, we are reporting two cases in which analysis of cfDNA was performed in patients with liver disease and with no sign of malignancy during the prior HCC screening by abdominal ultrasound, detecting mutations almost 1 year before imaging detection. Recently, detection at low frequency of HCC driver mutations (TP53, CTNNB1 and TERT promoter) has been reported in cfDNA of cirrhotic patients [35]. With our approach, we found that C228T TERT promoter mutation was detected at a frequency of 8% and 5.34% 12 and 10 months before diagnosis in patients VH341 and VH381, respectively. Nevertheless, this is exploratory and preliminary evidence, and further support to confirm the potential role of cfDNA in improving early diagnosis of HCC in at-risk patients is required. A multicentric study analyzing the levels of driver mutations as TERT [36,37,38] in the cfDNA of cirrhotic patients would be an appealing future approach to further develop the potential usefulness of cfDNA as a diagnostic biomarker.

Even after curative treatment, relapse remains a significant threat for many cancer patients, and it is difficult to detect minimal residual disease by imaging or tissue biopsy. Previous studies showed that ctDNA could be used for monitoring disease load, providing clinically relevant lead times compared to imaging techniques in colorectal cancer [39]. It has also been observed that cfDNA-positive patients are more likely to experience a relapse than the cfDNA-negative ones, showing a shorter disease-free survival [34,40]. Furthermore, it has been reported that cfDNA can be used to monitor dynamic changes in tumor burden, analyzing both genetic and epigenetic status, using minimally invasive blood sampling [41,42]. In addition, genetic analysis of cfDNA during clinical follow-up of patients could be useful in identifying the appearance of resistant subclones [43,44]. Another study investigated cfDNA and protein biomarkers in a long-term follow-up of patients with HCC, concluding that both SNVs and copy number variations (CNVs) possessed the capability to dynamically reflect HCC tumor burden [9]. In this study, we have observed that the somatic mutations on known HCC-related driver genes, such as as TERT, TP53 and CTNNB1 in cfDNA, were consistently and dynamically correlated with tumor burden during patient follow-up.

Genetic information from cfDNA could provide a tumor-specific molecular profile of tumors. This information could guide targeted therapy, improving the choice of the appropriate treatment for each patient. The half-life of cfDNA in the circulation is between 16 min and 2.5 h [45]; for this reason, cfDNA can be considered a real-time snapshot reflecting the molecular evolution of tumors [43]. Noninvasive access to molecular information allows real-time monitoring of treatment effectiveness in some type of tumors. Unfortunately, the most prevalent mutations in HCC, explored in the present study, are not therapeutic targets. Our approach, selecting the regions enriched with hotspot containing frequent mutations in TERT promoter, TP53, CTNNB1, AXIN1 and ARID1A, avoids the cost of performing more expensive techniques, such as whole exome or genome sequencing, which is unaffordable for the economic health system.

Analysis of cfDNA requires the evaluation of nontumor variant background noise. To achieve this aim, we included a control group of healthy patients, as well as PBMCs from each patient. Nonetheless, there are still many unknown aspects about the origin of variants in plasma and its biological meaning.

The main limitation of our study is its small sample size, primarily due to the unicentric nature of the study. This also led to the possibility that specific mutations as potential biomarkers for prognosis could not be identified. However, the potential prognostic value of cfDNA levels and number of mutations in plasma observed in our series deserves further investigation and validation in larger cohorts of patients. It is plausible to predict that cfDNA might play a major role in the near future in early diagnosis, prognostic estimation and management of HCC.

## 5. Conclusions

In conclusion, total cfDNA levels and detection of the most prevalent HCC mutations have prognostic implications that could refine the standard surveillance after curative treatment of early-stage HCC patients.

## Figures and Tables

**Table 1:** Univariate Cox analysis for mutation-related variables.

		Death			Recurrence		
Variable		HR	(95% CI)	*p*-Value	HR	(95% CI)	*p*-Value
Presence of mutations in HCC Tissue	No	1		0.939	1		0.802
	Yes	0.92	(0.11; 7.91)		0.82	(0.18; 3.82)	
Number of mutations in HCC Tissue		0.99	(0.82; 1.19)	0.886	1.03	(0.93; 1.14)	0.592
Number of mutated genes in HCC tissue		0.76	(0.36; 1.59)	0.465	1.11	(0.71; 1.73)	0.643
Presence of mutations in cfDNA	No	1		0.589	1		0.484
	Yes	0.55	(0.06; 4.91)		0.57	(0.12; 2.72)	
Number of mutations in cfDNA		**1.11**	**(1.02; 1.20)**	0.015 *	**1.16**	**(1.06; 1.27)**	0.001 *
Number of mutated genes in cfDNA		**2.37**	**(1.18; 4.74)**	0.015 *	**2.88**	**(1.52; 5.47)**	0.001 *
Number of Mutations/cfDNA^(1)^		1.07	(0.99; 1.16)	0.089	**1.2**	**(1.06; 1.35)**	0.003 *
Presence of mutations in Adj Tissue	No	1		0.158	1		0.9280
	Yes	0.21	(0.02; 1.85)		0.95	(0.30; 3.04)	
Number of mutations in Adj Tissue		0.97	(0.80; 1.17)	0.754	1.06	(0.98; 1.15)	0.1359
Number of mutated genes in HCC tissue		0.25	(0.03; 2.02)	0.193	1.17	(0.79; 1.72)	0.4285

Number of obs = 26; HCC: hepatocellular carcinoma, cfDNA: cell-free DNA. Data in bold mean the significant values. *: Statistically significant.

**Table 2:** Multivariate Cox regression analysis for mutation-related variables.

		Death			Recurrence		
Cut-Off Values		HR	(95% CI)	*p*-Value	HR	(95% CI)	*p*-Value
Number of mutations in HCC Tissue	No (<6)	1		0.4796	1		0.346
	Yes (>6)	2.23	(0.24; 20.4)		2.09	(0.45; 9.74)	
Number of mutations in cfDNA	No (<4)	**1**		0.0078 *	1		0.06
	Yes (>4)	**11.66**	**(1.91; 71.2)**		3.54	(0.94;13.35)	
Number of mutated genes in cfDNA	No (<2)	**1**		0.0287 *	**1**		0.009 *
	Yes (>2)	**5.31**	**(1.19; 23.77)**		**9.61**	**(1.75; 52.7)**	
N Mutations/cfDNA	No (<6)	1		0.051	**1**		0.007 *
	Yes (>6)	7.07	(0.99; 50.5)		**7.44**	**(1.71; 32.3)**	

HCC: hepatocellular carcinoma, cfDNA: cell-free DNA. Data in bold mean the significant values. *: Statistically significant.

## Data Availability

Raw sequencing data from samples included in this article will be openly available upon publication via Sequence Read Archive of the NCBI (Accession number PRJNA791805).

## Supplementary Materials

The following supporting information can be downloaded at: https://www.mdpi.com/article/10.3390/cancers14163875/s1, Figure S1: Dynamical changes in cfDNA levels and mutational load in patient VH343 and VH369; Figure S2: Dynamical changes and mutational load in patient VH371, Table S1: Primer design for sequencing, Table S2: Clinicopathological characteristics in HCC patients, Table S3: Clinicopathological characteristics in BCLC stage patients, Table S4: Somatic mutations identified, Table S5: Univariate Cox analysis of independent risk factors of survival and recurrence, Table S6:ddPCR TERT.

## Abbreviations

cfDNA: cell-free DNA; HCC: hepatocellular carcinoma; MAFLD: metabolic-associated fatty liver disease. AFP: alpha-fetoprotein, PBMC: peripheral blood mononuclear cells, SNV: single-nucleotide variants, CNV: copy number variations.
