# Supplementary material for: Ultra Deep Sequencing of Circulating Cell-Free DNA as a Potential Tool for Hepatocellular Carcinoma Management
Source: Cancers (Basel). 2022 Aug 11;14(16):3875. doi: 10.3390/cancers14163875 (PMC9406074; doi:10.3390/cancers14163875)
Supplement: Supplementary file 1 [file cancers-14-03875-s001.zip › Supplementary Data_Higuera et al.pdf]

Supplementary Information:

Materials and Methods

Digital PCR

C228T TERT mutation was analyzed by QuantStudio 3D Digital PCR System (Thermo Fisher, USA), with TaqMan primers and probes also from Thermo Fisher (Hs000000092_rm). A known positive and negative DNA was included as control in each ddPCR run. The PCR reaction was performed using 1X ddPCR Master Mix, 1X TaqMan primer/probe for C228T TERT mutation, and 6,13 ul of DNA to a final reaction volume of 14 μL. The PCR mixture was loaded into the 20K Chip v2. The cycling condition were 96°C for 10 min, followed by 54 cycles of 55°C for 2 min and 98°C for 30 s, and 1 cycle of 55°C for 2 min and 10oC hold indefinitely. The analysis and quantification of wild type and mutated alleles were performed using the QuantStudio 3D AnalysisSuite Cloud Software from Thermo Fisher.

Supplementary Table S1: Primer design for sequencing.

Gene name		Primer sequence	5' Genome co-ordenates	3' Genome co-ordenates	Size	Tm	Size amplicon	Size amplicon +MID
**M13**	FW	GTTGTAAAACGACGGCCAGT			20	60		
	RV	CACAGGAAACAGCTATGACC			20			
**ARID1A**	FW	TTATGGCACACTCCCTCCAG	26763119	26763100	20	59	157	197
	RV	CATGGCGACAGCAGTTTCTT	26763256	26763237	20			
**AXIN1**	FW	CGGATCTGGACCTGGGGTAT	346835	346816	20	57	151	191
	RV	AAGTCGGCACAGCCCTCCTG	346685	346704	20			
**CTNNB1**	FW	CAGAAAAGCGGCTGTTAGTCA	41224583	41224563	21	54	145	185
	RV	CTCATACAGGACTTGGGAGGT	41224707	41224687	21			
**TP53**	FW	TGGGCCTGTGTTATCTCCTA	7674311	7674292	20	57	148	188
	RV	GGCAAGTGGCTCCTGACCT	7674182	7674164	19			
**TP53**	FW	AAGATGTTTTGCCAACTGGC	7680753	7680734	20	57	166	206
	RV	CATCGCTATCTGAGCAGCG	7680899	7680881	19			
**TERT**	FW	CAGCGCTGCCTGAAACTC	1295036	1295053	18	65	163	203
	RV	GTCCTGCCCCTTCACCTT	1295198	1295181	18			

Abbreviations: ARID1A: AT-Rich Interaction Domain 1A gene, AXIN1: Axis Inhibition Protein 1 CTNNB1: Catenin beta 1, TP53: tumor protein p53, TERT, telomerase reverse transcriptase. FW: Forward primer, Rv: Reverse primer, Tm: Melting Temperature

Supplementary table S2. Clinicopathological characteristics in HCC patients (n=30) and Controls (n=10)

**Demographics**		**Cases**
**Clinicopathological characteristics**		
Gender, n (%)	Male	23 (76.6%)
Age, median (range)		61.5 (86-20)
Race/Ethnicity, n (%)	Caucasian	25 (83.3%)
Etiology, n (%)	HCV	11 (36.6%)
	MAFLD	5 (16.6%)
	Alcohol	4 (13.3%)
	HBV	2 (6.6%)
	Others	8 (26.6%)
Fibrosis (n (%)	F4	8 (26.6%)
	F3	9 (30%)
	F2/F1	13 (43.3%)
BCLC	BCLC 0	5 (16.7%)
	BCLC A	25 (83.3%)
Tumor size (cm), median (range)		3.75 (1-12)
Single tumor (%)	Yes	27 (90%)
	No	3 (10%)
Microvascular invasion (%)	Absent	24(80%)
	Present	6 (20%)
Satellites (%)	Absent	26 (86.7%)
	Present	4 (13.3%)
Tumor Differentiation	Well	5 (16.7%)
	Moderate	22 (73.3%)
	Poor	3 (10%)
Events (%)	Recurrence	12 (40%)
	Death	7 (23.3%)
Follow-up, median months (range)		22.5 (1-50)
**Laboratory values**		
AFP (mg/dL), median (range)		17 (1.3-3233)
Bilirubin (mg/dL), median (range)		0.77 (0.37-2.25)

Albumin (g/L), median (range)		4.1 (2.5-4.7)
Platelet count (), median (range)		193 (103-266)

**Demographics (Control samples)**		**Cases**
**Clinicopathological characteristics**		
Gender, n (%)	Male	(7) 70%
Age, median (range)		56.5 (50-62)

HCV:Hepatitis C virus, MAFLD: Metabolic associated fatty liver disease, HBV: Hepatitis B virus, AFP: Alpha-fetoprotein

Supplementary table S3. Clinicopathological characteristics in HCC patients with different BCLC stages (n=51)

**Demographics**					
**Clinicopathological characteristics**					
		**BCLC 0/A**	**BCLC B**	**BCLC C**	**BCLC D**
**Gender, n (%)**	**Male**	23 (76.6%)	5 (71.4%)	7 (87.5%)	4 (66.6%)
**Age, median (range)**	**Years**	61.5 (86-20)	73.2 (83-55)	71 (82-51)	75 (85-60)
**Race/Ethnicity, n (%)**	**Caucasian**	25 (83.3%)	7 (100%)	8 (100%)	6 (100%)
**Etiology, n (%)**	**HCV**	11 (36.6%)	4 (57.1%)	2 (25%)	4 (66.7%)
	**MAFLD**	5 (16.6%)	1 (14.3%)	0 (0%)	0 (0%)
	**Alcohol**	4 (13.3%)	1 (14.3%)	3 (37.5%)	1 (16.7%)
	**HBV**	2 (6.6%)	1 (14.3%)	2 (25%)	0 (0%)
**Tumor size (cm), median (range)**	**Rx**	3.75 (12-1)	4.3 (6.5-1.6)	NM 75% Diff/MF	NM 83.3% Diff/MF
**Single tumor n(%)**	**Rx**	27 (90%)	3 (42.9%)	2 (25%)	1 (16.7%)
**Follow-up, median (range)**	**Months**	22.5 (1-50)	18 (0-51)	3.6 (0-9)	0.63 (0-26)
**Laboratory values**					
**AFP, median (range)**	**(mg/dL)**	17 (1.3-3233)	8.1 (2.7-2733)	117.9 (3.1-19283)	701.25 (2.5-1400)
**Bilirubin, median (range)**	**(mg/dL)**	0.77 (0.37-2.25)	1 (1.76-2.15)	1.46 (0.39-3.92)	0.91 (0.49-3.89)
**Albumin, median (range)**	**(g/L)**	4.1 (2.5-4.7)	4.2 (2.2-4.7)	3 (2.7-4.1)	2.55 (2-4.1)
**Platelet count, median (range)**		193 (103-266)	143 (76-173)	162 (97-310)	194 (89-385)

HCV:Hepatitis C virus, MAFLD: Metabolic associated fatty liver disease, HBV: Hepatitis B virus, NM: Non measurable Diff: Difusse, MF:Multifocal, Rx: Radiological data, AFP: Alpha-fetoprotein

Supplementary Table S5: Univariate Cox analysis of independent risk factors of survival and recurrence

	Survival			Recurrence		
**VARIABLE**	**HR**	**(95%CI)**	**p-Value**	**HR**	**(95%CI)**	**p-Value**
Age	0.97	(0.93; 1.01)	0.184	0.998	(0.96; 1.03)	0.8993
Gender	0.99	0.11; 8.47)	0.990	2.04	(0.55; 7.59)	0.2879
cfDNA ng/ml	1.16	(0.76; 1.77)	0.501	0.91	(0.63; 1.31)	0.6147
Size main tumor	1.000	(0.77; 1.29)	0.999	**1.23**	**(1.05; 1.45)**	**0.0109***
N of nodules (single *vs* multiple)	1.75	(0.19;15.72)	0.883	2.74	(0.56;13.46)	0.2189
Glypican [1]	0.54	(0.06; 4.96)	0.589	0.27	(0.05; 1.43)	0.1248
Vascular Invasion [2]	1.15	(0.13;10.35)	0.903	1.09	(0.23; 5.06)	0.9160
Bilirrubin	6.50	(0.84;50.13)	0.072	2.21	(0.41;11.89)	0.3570
Albumin (g/L)	0.58	(0.19; 1.77)	0.335	0.88	(0.34; 2.27)	0.7865
Creatinin	0.13	(0.00; 9.74)	0.354	0.10	(0.00; 2.53)	0.1627
AFP(ng/mL) [2]	1.000	(0.97; 1.02)	0.971	1.005	(0.99; 1.01)	0.2679
Platelets	1.000	(0.98; 1.01)	0.935	1.004	(0.99; 1.01)	0.2667

1: number of obs=21, 2: number of obs=28, cfDNA: cell free DNA

cfDNA: Cell Free DNA, AFP:Alpha-fetoprotein

Supplementary Table S6: Validation of C228T TERT mutation by ddPCR

Sample	Tissue	% Freq ddPCR	% Freq NGS
VH335	HCC	34.63	66.95
VH335	Adj	0.285	9.47
VH335	cfDNA	8.17	76.84
VH337	HCC	51.68	66.11
VH337	Adj	0.562	1.13
VH337	cfDNA	5.317	5.83
VH339	HCC	43.628	66.77
VH339	Adj	0.828	1.86
VH339	cfDNA	2.3	59.46
VH341	HCC	0.348	1.95
VH341	cfDNA	8.74	10.48
VH344	Adj	0.00664	1.12
VH344	cfDNA	0.844	54.02
VH345	HCC	9.316	28.4
VH345	Adj	1.207	1.7
VH345	cfDNA	14.687	23
VH347	HCC	32.256	71.3
VH347	Adj	0.839	2.2
VH349	HCC	20.702	52.3
VH349	cfDNA	4.4	12.9
VH351	HCC	34.71	46.5
VH351	Adj	0.144	1
VH351	cfDNA	1.745	1.2
VH353	cfDNA	1.67	10.14
VH355	HCC	41.912	74.3
VH355	Adj	0.239	1.8
VH369	HCC	44.55	51
VH369	Adj	0.419	2.68
VH369	cfDNA	7.064	4.59
VH371	HCC	76.014	69.26
VH371	cfDNA	1.525	1.54
VH373	HCC	22.987	28.97
VH373	cfDNA	2.757	2.24
VH375	HCC	0.0385	1.47
VH375	cfDNA	3.403	3.47
VH377	HCC	1.823	3.29
VH377	Adj	0.271	2.36
VH377	cfDNA	1.313	1.85
VH381	HCC	32.887	37.72
VH381	cfDNA	3.052	38.4

B1921	HCC	29.1	39.16
B1921	cfDNA	35.594	43.37
B1932	HCC	3.265	1.08
B1932	cfDNA	23.757	34.23
B1933	cfDNA	29.754	33.61
VH383	cfDNA	4.954	3.85
VH391	cfDNA	3.018	5.53
VH393	HCC	19.83	33.94
VH393	HCC	1.39	1.82
VH401	HCC	22.376	17.2
VH411	HCC	42.085	53
VH411	Adj	0.0529	1.33
VH411	cfDNA	2.537	1.91
VH413	HCC	54.979	33.59
VH417	HCC	26.707	27.91
VH417	Adj	8.686	3
VH417	cfDNA	0.239	0.86
VH427	cfDNA	1.456	2.85

HCC: Hepatocellular Carcinoma Tissue. Adj: Adjacent Tissue. cfDNA: Cell free DNA.

Supplementary Figure S1

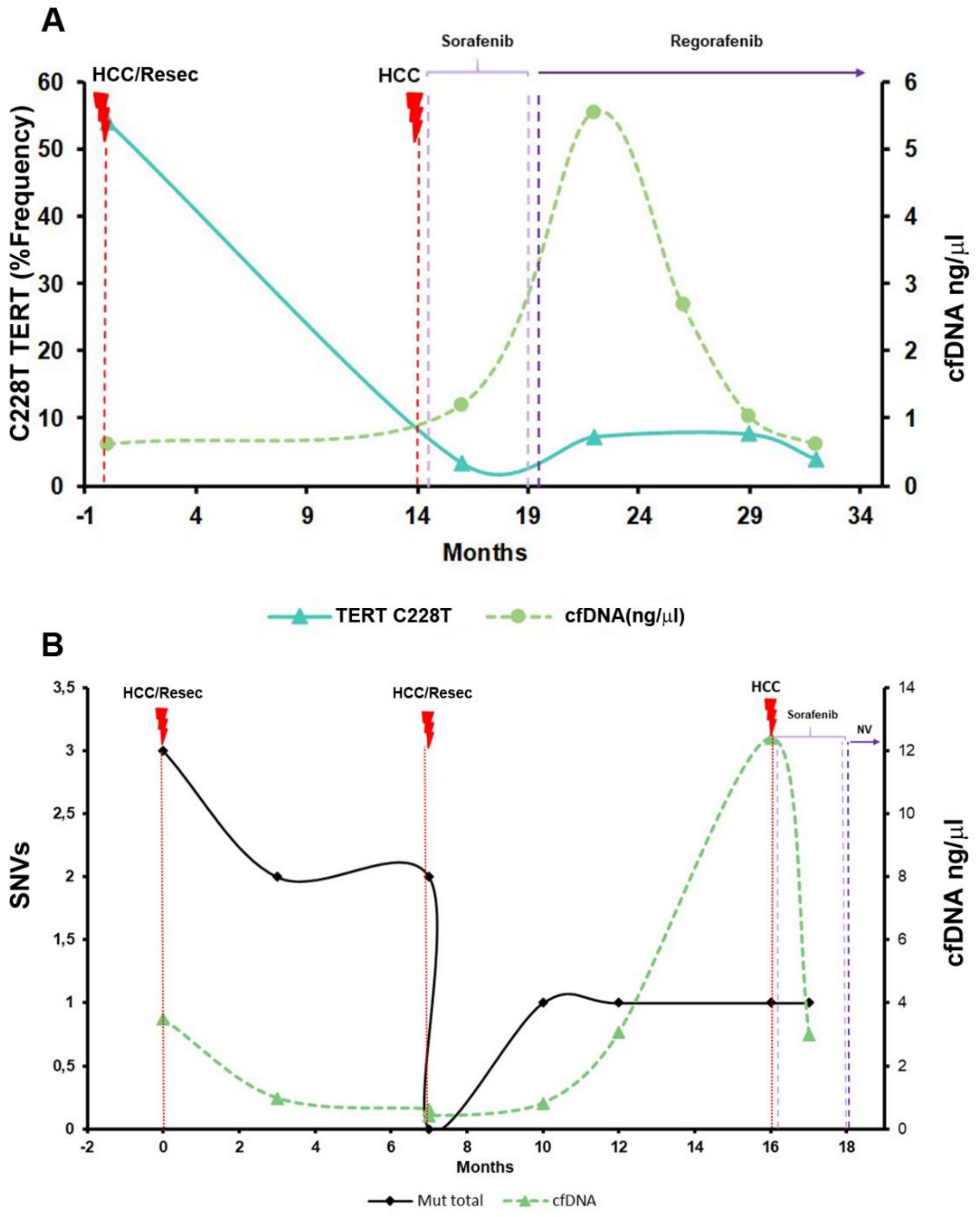

Figure S1. **A**: Dynamical changes in cfDNA levels and C228T (-124) TERT mutations frequency correlating with HCC progression in Patient VH343 along 33 months of follow-up. **B**: Dynamical fluctuations in cfDNA levels and absolute mutational load correlating with HCC progression along the 18 months of follow-up in Patient VH369. HCC/Resec: Detection and resection of HCC, NV: Systemic treatment with Nivolumab, cfDNA: Cell-free DNA, SNVs: Single nucleotide variant.

Supplementary Figure S2

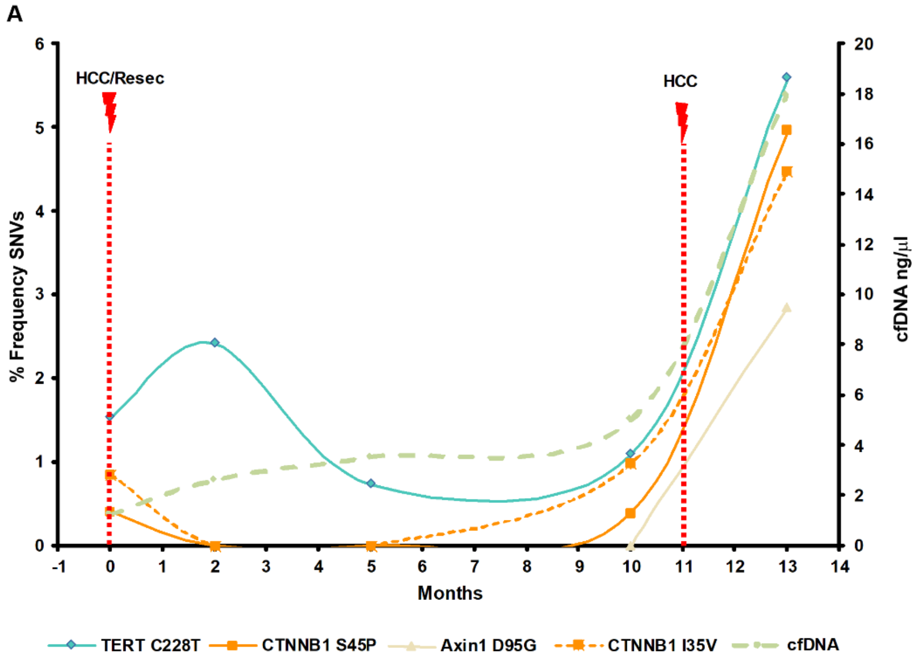

Figure S2. **A**: Dynamical changes in cfDNA levels and TERT, CTNNB1 and AXIN1 mutations frequency correlating with HCC progression in Patient VH371 along 13 months of follow-up. HCC/Resec: Detection and resection of HCC, cfDNA: Cell-free DNA, SNVs: Single nucleotide variant.
